# A novel pseudovirus‐based mouse model of SARS-CoV-2 infection to test COVID-19 interventions

**DOI:** 10.1186/s12929-021-00729-3

**Published:** 2021-04-30

**Authors:** Ssu-Hsueh Tseng, Brandon Lam, Yu Jui Kung, John Lin, Li Liu, Ya Chea Tsai, Louise Ferrall, Richard B. S. Roden, T. C. Wu, Chien-Fu Hung

**Affiliations:** 1 Department of Pathology, Johns Hopkins School of Medicine, Baltimore, MD USA; 2 Graduate Program in Immunology, Johns Hopkins School of Medicine, Baltimore, MD USA; 3 Department of Oncology, Johns Hopkins School of Medicine, Baltimore, MD USA; 4 Department of Obstetrics and Gynecology, Johns Hopkins School of Medicine, Baltimore, MD USA; 5 Molecular Microbiology and Immunology, Johns Hopkins School of Medicine, Baltimore, MD USA; 6 Johns Hopkins University School of Medicine, 1550 Orleans Street, CRBII 307, Baltimore, MD 21287 USA

**Keywords:** SARS-CoV-2, COVID-19, Pseudovirus, Adenovirus

## Abstract

**Background:**

The spread of SARS-CoV-2, the virus that causes Coronavirus Disease 2019 (COVID-19), has been characterized as a worldwide pandemic. Currently, there are few preclinical animal models that suitably represent infection, as the main point of entry to human cells is via human angiotensin-converting enzyme 2 (ACE2) which is not present in typical preclinical mouse strains. Additionally, SARS-CoV-2 is highly virulent and unsafe for use in many research facilities. Here we describe the development of a preclinical animal model using intranasal administration of ACE2 followed by non-infectious SARS-CoV-2 pseudovirus (PsV) challenge.

**Methods:**

To specifically generate our SARS-CoV-2 PsV, we used a lentivirus system. Following co-transfection with a packaging plasmid containing HIV Gag and Pol, luciferase-expressing lentiviruses, and a plasmid carrying the SARS-CoV-2 spike protein, SARS-CoV-2 PsVs can be isolated and purified. To better understand and maximize the infectivity of SARS-CoV-2 PsV, we generated PsV carrying spike protein variants known to have varying human ACE2 binding properties, including 19 deletion (19del) and 19del + D614G.

**Results:**

Our system demonstrated the ability of PsVs to infect the respiratory passage of mice following intranasal hACE2 transduction. Additionally, we demonstrate in vitro and in vivo manipulability of our system using recombinant receptor-binding domain protein to prevent PsV infection.

**Conclusions:**

Our PsV system is able to model SARS-CoV-2 infections in a preclinical mouse model and can be used to test interventions or preventative treatments. We believe that this method can be extended to work in various mouse strains or to model infection with different coronaviruses. A simple in vivo system such as our model is crucial for rapidly and effectively responding to the current COVID-19 pandemic in addition to preparing for future potential coronavirus outbreaks.

**Supplementary Information:**

The online version contains supplementary material available at 10.1186/s12929-021-00729-3.

## Introduction

Since December 2019, when the World Health Organization first cited the disease, SARS-CoV-2 has spread to 219 countries. Consequently, Coronavirus Disease 2019 (COVID-19) has been characterized as a pandemic. As of November 2020, 47 Million cases and 1.2 million deaths have been reported worldwide [1, 2]. The combined high-level transmissibility and virulence of SARS-CoV-2 underscore the urgent need to develop novel, effective, and broadly applicable strategies to prevent and manage SARS-CoV-2 infection.

The severe acute respiratory syndrome-coronavirus-2 (SARS-CoV-2) is the causative agent of COVID-19. SARS-CoV-2, as well as other related coronavirus family members, utilize host angiotensin-converting enzyme 2 (ACE2) as the binding and entry receptor [3]. The SARS-CoV-2 spike protein (S) comprises two functional domains: an S1 trimer and S2 stalk [4]. The receptor-binding domain (RBD) of SARS-CoV-2, as with SARS-CoV, permits binding to ACE2, and is located in S1 [5]. Viral tropism of SARS-CoV-2 depends on tissue expression and distribution of ACE2, which is primarily of importance in airway epithelial cells where high levels of ACE2 expression occurs depending on cell differentiation status [6]. Upon binding and infection, the virus replicates, leading to the manifestation of a myriad of symptoms in approximately 20 % of patients, including fever, cough, respiratory distress, and loss of smell [1, 2].

Due to the critical role of the RBD in SARS-CoV and SARS-CoV-2, it has been explored extensively as a major prophylactic and therapeutic viral target [5]. Currently, in the SARS-CoV-2 vaccine development landscape, inactivated, live attenuated, recombinant protein, virus-like particle (VLP), vectors, inactivated virus, DNA, and RNA strategies are being explored [7]. This includes numerous products in phase III trials, 2 replication-incompetent vector-based vaccines approved without phase III trial studies in Russia and China, and 2 RNA-based vaccines being administered in teh United States [7]. The area is rapidly evolving with new products and formulations arising almost daily.

A major roadblock in developing vaccines for SARS-CoV-2 is an appropriate animal model selection for preclinical efficacy and safety studies. The reasons for this are two-fold; SARS-CoV-2 cannot infect laboratory mice, the most widely used animal system in biomedical studies, due to incompatibility of mouse ACE2 and SARS-CoV-2 RBD, and practical challenges of using SARS-CoV-2, an animal biosafety level 3 (ABSL-3) pathogen, because most organizations lack the necessary facilities and trained personnel.

To specifically tackle the incompatibility of infection in murine systems, numerous strategies have been employed [8–10]. These approaches seek to introduce human ACE2 into mice. For example, mice transgenic for human ACE2 under the K18 human keratin promoter have been developed, which express ACE2 in the epithelia, including in the airway [11]. While convenient, this system does not allow the flexibility to move readily between mouse strains and restricts the ability to investigate infectivity among other gene alterations without crossbreeding. Other strategies have used adenovirus transduction, or other viruses, to express human ACE2 in the airway following intranasal exposure (AdV-hACE2) [9] and permit infection with SARS-CoV-2 under ABSL-3 conditions to model human infection.

In this study, we utilize a pseudovirus (PsV) strategy to model SARS-CoV-2 infection. A primary benefit of this system is that it can be fully employed in widely available ABSL-2 conditions, and without placing laboratory personnel at significant risk. To specifically generate our SARS-CoV-2 PsV, we used a lentivirus system. Lentiviruses belong to the *retroviridae* family and have been widely used in the generation of PsV. Following co-transfection with a packaging plasmid containing HIV Gag and Pol, luciferase-expressing lentivirus, and a plasmid carrying the SARS-CoV-2 spike protein, SARS-CoV-2 PsV can be isolated and purified. To better understand and maximize the infectivity of SARS-CoV-2 PsV, we generated PsV carrying spike protein variants known to have varying human ACE2 binding properties, including 19 deletion (19del) and 19del + D614G. Both of these variants have been demonstrated to have significantly higher PsV infection and replication potential. Specifically, the 19del was demonstrated to permit significantly higher infection of SARS-CoV PsVs, and the D614G mutation emerged early in the COVID-19 pandemic, showed heightened infection potential, and quickly became the most common variant spreading [12, 13].

## Materials and methods

### Mouse experiments

6-8-week-old female BALB/c were purchased from Charles River Laboratories (Frederick, Maryland, USA). All mice were maintained under specific pathogen-free conditions at the Johns Hopkins University School of Medicine Animal Facility (Baltimore, Maryland, USA). Recombinant adenoviral vectors expressing human ACE2 (AdV-hACE2) were purchased from the University of Iowa Viral Vector Core. To minimize animal suffering, all the procedures were performed under anesthesia by intramuscular injection of ketamine. For PsV infection, mice were first treated with 2.5 × 10^8^ PFU of AdV-hACE2 via intranasal (i.n.) administration. Five days after AdV transduction, mice were treated with 850ng of WT, 19del, or 19del + D614G PsV i.n. In vivo bioluminescence imaging was performed using the IVIS Series 2000 (PerkinElmer). The mice were treated with 200ug of D-luciferin (GoldBio) via intranasal administration. 5 min after D-luciferin administration, mice were imaged on the IVIS Spectrum for 5 min. To quantify the luminescence signals, the nose region in the displayed images were quantified as total photon counts using Living Image 3.0 Software (Xenogen). To determine Lung infection, mice were treated with D-luciferin intranasally and intraperitoneally. 5 min after D-luciferin administration, mice were sacrificed, and lungs were harvested for imaging. For in vivo blocking experiments, mice were pre-treated with 30ug of RBD-His protein i.n. one hour before PsV infection. Mice were imaged on day 4.

### Plasmids

 To generate the 19del pseudovirus (PsV) plasmid (PCMV3-S-cd19), 19 amino acids were deleted in the spike protein sequence of SARS-CoV-2 strain Wuhan-Hu-1. PCR fragment was amplified by primers TTTGGTACCATGTTTGTGTTCCTGGTGC and AAATCTAGATTAACAACAGGAGCCACAGGAA and template (pCMV3-SARS-CoV-2-S, Sino Biological Inc. Beijing, China) and cloned into PCMV3 vector. To generate 19del + D614G mutation PsV plasmid (PCMV3-S614-cd19), site-directed mutagenesis was used for generating D614G mutation in the SARS-CoV-2 spike sequence. PCR fragment was amplified by primers (TAATACGACTCACTATAGGG, GGCACCTCAGTACAGTTCACACCCTGGTAGAGCACAGCCACC, GGTGGCTGTGCTCTACCAGGGTGTGAACTGTACTGAGGTGCC, and AAATCTAGATTAACAACAGGAGCCACAGGAA) and template (pCMV3-S-cd19) and cloned into PCMV3 vector.

### Generation of SARS-CoV-2 spike PsV

We constructed a SARS-CoV-2 spike PsV using a lentivirus packaging system. 293TT cells [14] were co-transfected with a packaging plasmid, CMV∆8.91, expressing Gag and Pol, a lentivirus vector expressing luciferase, pCDH1puro-LucGFP, and an expression plasmid with PCMV3-S-cd19 or PCMV3-S614-cd19. All plasmids were at 1.0 µg/ml and used in the following ratios: pCDH1puro-Luc, 9 µl; pCMV∆8.91, 12 µl; and PCMV3-S-cd19 or PCMV3-S614-cd19, 3 µl. After 3 days of incubation at 37 °C in Opti-M medium, cells were harvested and pelleted at 2000g **×** 5 min. The supernatant was filtered through a 0.45 μm filter, with/or without concentration using an Amicon Ultra-15 filter unit, aliquoted, and stored at − 80 °C. The amount of PsV was quantified by HIV-1 Gag p24 DuoSet ELISA kit (R&D Systems) following the manufacturers’ instructions.

### In vitro pseudovirus infection

 To test the activity of PsVs, 25ng (HIV1 Gag p24) of PsV was added to 10^4^ 293TT cells stably transfected with hACE2 receptor SARS-CoV-2, in the well of a 96-well plate. After incubation for 72 h, BPS Bioscience one step Luciferase Reagent buffer and Luciferase Reagent substrate were added and luciferase activity measured in a luminometer. For blocking experiments, 10ug/ml of SARS-CoV-2 Spike RBD-His Recombinant Protein (Sino Biological Inc.) was added to hAEC2-expressing 293TT cells and incubated at 37 **°**C for 1 h. One hour after RBD blocking, PsV was added to the RBD pre-treated hAEC2-expressing 293TT cells and incubated for 72 h.

## Results

### Mice infected intranasally with SARS-CoV-2 pseudovirus post AdV-hACE2 demonstrate signs of infection

**Fig. 1:**
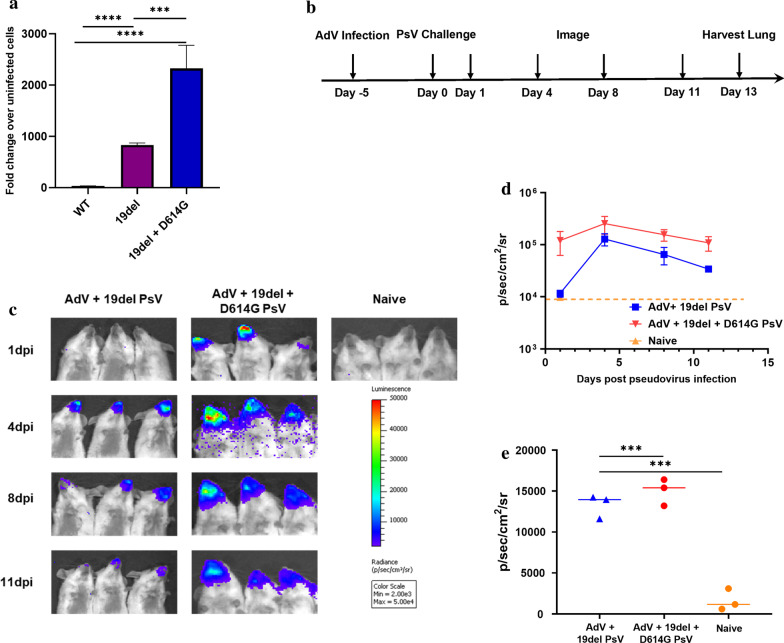
Development of a pseudovirus model of SARS-CoV-2 infection. Infectivity of wild-type (WT), 19del, and 19del + D614G SARS-CoV-2 pseudoviruses. hACE2 expressing 293TT cells were infected with wild-type, 19del, or 19del + D614G SARS-CoV-2 pseudoviruses (quantified as 25ng of HIV Gag p24 protein) for 72 h. Cell lysates were collected and relative luminescence units (RLU) were measured (**a**). Schematic diagram of in vivo infection protocol (b). 6–8-week-old female BALB/c mice were infected intranasally (i.n.) with a hACE2-expressing adenovirus (2.5 × 10^8^ PFU) (AdV-hACE2). Five days later, mice were challenged with luciferase-expressing pseudoviruses (n = 3 per group) (quantified as 850ng of HIV Gag p24 protein). Mice were imaged on days 1, 4, 8, and 11 after pseudovirus infection. Untreated naïve mice (n = 3) were used as background controls. Representative bioluminescence and kinetics of mouse infection with 19del or 19del + D614G pseudoviruses **(c)**. Quantification of bioluminescence signal in the nasal passage **(d)** and lungs (collected 13 days post infection) **(e)**. Blocking SARS-CoV-2 PsV infection through RBD treatment. hAEC2-expressing 293TT cells were pre-treated with RBD (1ug/ml) one hour before pseudovirus challenge. Data expressed as mean ± SEM. Student’s t test used to analyze differences between groups. *p < 0.05, **p < 0.01, ***p < 0.001, ****p < 0.0001

We first sought to assess the ability of our SARS-CoV-2 PsVs to infect cells expressing hACE2. 293TT cells expressing hACE2 were infected with WT, 19del, or 19del + D614G PsVs (normalized for 25ng Gag protein). 72 h later, cells were lysed, and luminescence activity was measured using a luminometer. Compared to uninfected cells, we observed that WT SARS-CoV-2 PsVs exhibit a ~ 30-fold increase in relative luminescence units (RLU) (Fig. 1a), indicating that substantial infection occurred. Comparatively, both the 19del and 19del + D614G variant PsVs yielded a significantly higher degree of infection, 830- and 2325-fold increase over uninfected, respectively (Fig. 1a). Given this, we further pursued the infection potential of 19del and 19del + D614G PsVs in vivo.

To confer epithelial expression of hACE2 in BALB/c mice, we utilized the previously described AdV-hACE2 system [9]. Five days prior to PsV challenge, mice were infected intranasally (i.n.) with AdV-hACE2. Following this, mice were administered equal amounts of either 19del or 19del + D614G PsVs i.n. Infection was monitored by IVIS imaging at the indicated times (Fig. 1b). Consistent with our in vitro findings, infection was detected in both 19del and 19del + D614G PsV infected mice (Fig. 1c), with significantly higher levels of infection observed following 19del + D614G PsV infection (Fig. 1d). In addition, to assess the infection potential of our PsVs in the lower respiratory tract following i.n. challenge, we collected the lungs of mice and performed IVIS imaging. Similar to the nasal region, PsV infection was observed in the lungs (Additional file 1: Fig. S1a), with significantly higher levels of infection observed in 19del + D614G PsV infected mice (Fig. 1e).

### SARS-CoV-2 PSVs may serve as an attractive platform to test SARS-CoV-2 infections involving hACE2 in vitro

**Fig. 2:**
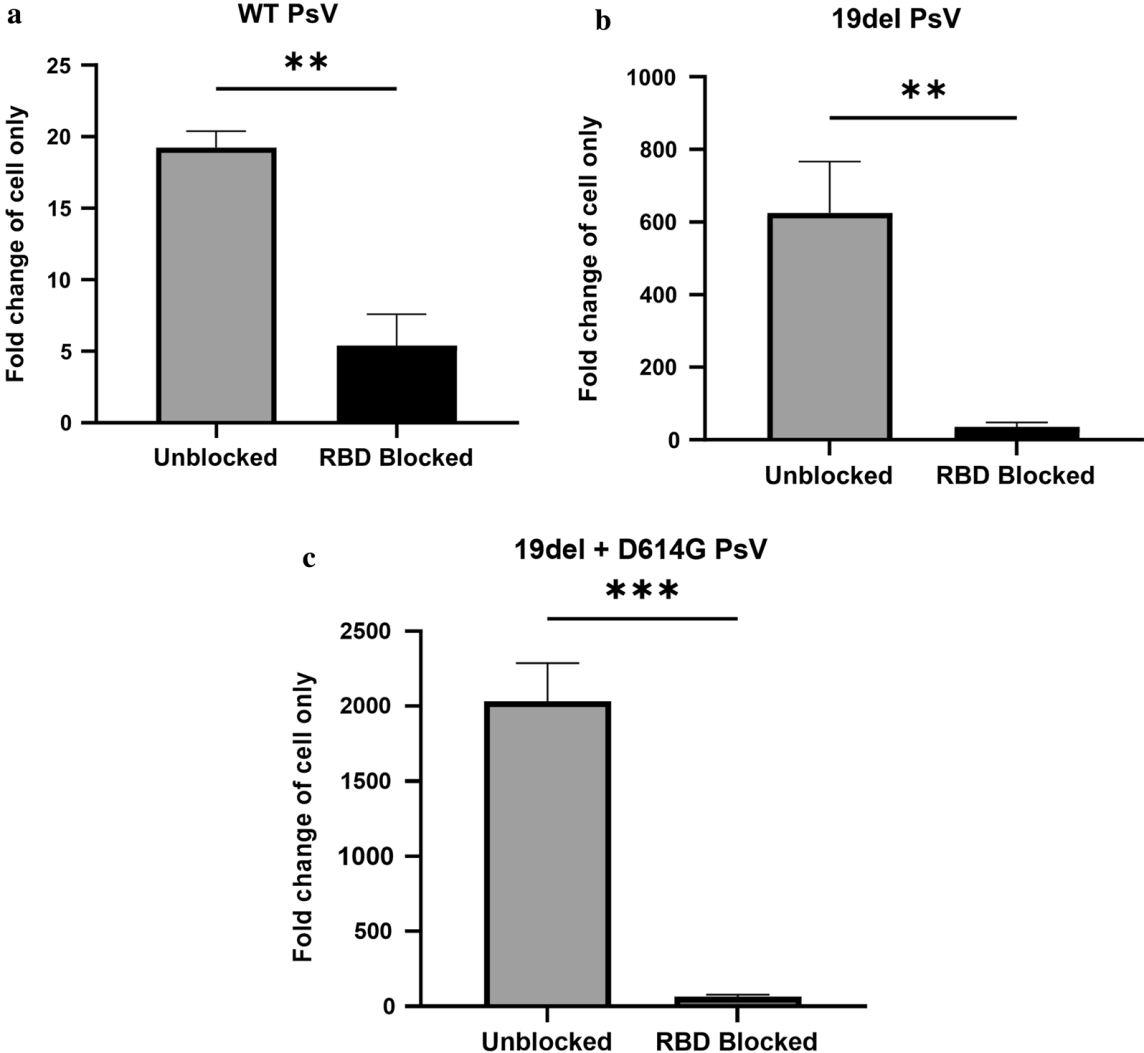
Efficacy of blocking SARS-CoV-2 PsV infection using soluble RBD in vitro. Ability of RBD to block WT **(a)**, 19del **(b)**, or 19del + D614G **(c)** pseudovirus infection. Following preincubation with RBD protein, hACE2 expressing 293TT cells were infected with WT, 19del, or 19del + D614G SARS-CoV-2 pseudoviruses (quantified as 25ng of HIV Gag p24 protein). 72 h later, cells were harvested and RLU was determined as a readout for infection. Data expressed as mean ± SEM. Student’s t test used to analyze differences between groups. *p < 0.05, **p < 0.01, ***p < 0.001, ****p < 0.0001

Given the substantial infectivity of 19del and 19del + D614G SARS-CoV-2 PsVs we observed in vitro and in vivo, we sought to explore the model’s potential to test SARS-CoV-2 interventions. An attractive approach is through the administration of viral attachment inhibitors, such as soluble RBD [15] which would bind hACE2 and block binding sites for SARS-CoV-2. We aimed to test this approach’s potential to limit WT, 19del, and 19del + D614G PsV infection in our model. 293TT cells expressing hACE2 were pretreated with RBD protein one hour prior to WT, 19del, or 19del + D614G PsV infection. 72 h later, cell lysates were collected and RLU was collected. RBD treatment successfully lowered the infection level of WT, 19del, and 19del + D614G PsVs in vitro, as significantly lower luciferase activity occurred upon RBD addition (Fig. 2a–c) in a concentration-dependent manner (Additional file 2: Fig. S2a).

### In vivo blocking of SARS-CoV-2 PsV infection using RBD protein blocking

**Fig. 3:**
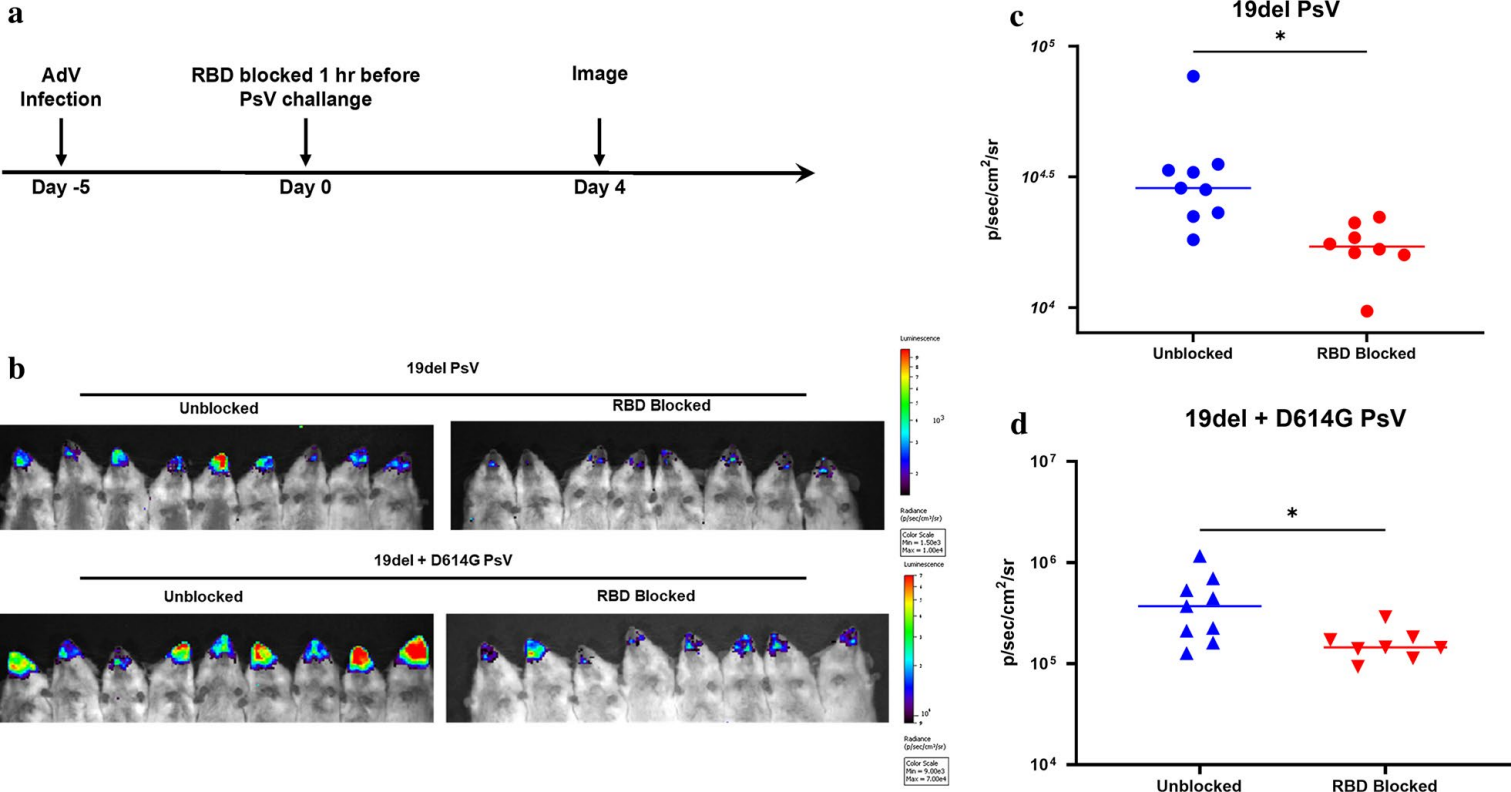
Proof-of-principle reduction of SARS-CoV-2 PsV infection in vivo following RBD protein administration. Schedule of infection and RBD blocking **(a)**. BALB/c mice were infected with AdV-hACE2 intranasally. Five days later, mice were treated with 30ug of RBD i.n. one hour before 19del or 19del + D614G pseudovirus infection. On Day 4 post-infection, mice were imaged for bioluminescence activity. Representative bioluminescence images of mice infected with 19del or 19del + D614G pseudovirus with or without RBD blocking **(b)**. Quantification of bioluminescence signal of mice infected with 19del **(c)** or 19del + D614G **(d)** pseudovirus with or without RBD blocking showing significantly lower infection in mice treated with RBD protein compared to unblocked control. Data expressed as mean ± SEM. Student’s t test used to analyze differences between groups. *p < 0.05, **p < 0.01, ***p < 0.001, ****p < 0.0001

Finally, to explore the potential of RBD blocking to limit PsV infection in vivo, we introduced AdV-hACE2 into BALB/c mice as in Fig. 1b and c. Prior to 19del or 19del + D614G SARS-CoV-2 PsV infection, mice were treated intranasally with RBD (Fig. 3a). Infectivity was monitored four days later. Similar to our in vitro findings, RBD blocking significantly reduced the ability of both variant PsVs to infect hACE2-expressing mice as compared to unblocked mice (Fig. 1b-d). This finding serves as a proof-of-principle study demonstrating the ability to interfere with SARS-CoV-2 PsV infection in vivo.

## Discussion

In summary, our results demonstrate the ability of Lenti-based SARS-CoV-2 PsVs to model SARS-CoV-2 infection following transduction with adenoviruses expressing hACE2. Current research on SARS-CoV-2 and other similar human coronaviruses, has been hindered due to the lack of easily reproducible preclinical mouse models. Our easy-to-use model of employs AdV to introduce hACE2 to the mouse respiratory system followed by i.n. administration of 19del or 19del + D614G SARS-CoV-2 pseudoviruses. In addition to representing an easily accessible mouse model, this method also circumvents the problem of using highly infectious coronaviruses requiring ABSL-3 level labs.

This model system can be established in vitro using hACE2 expressing cell lines easily test viral infection inhibitors. In vivo, following transduction with AdV-hACE2, PsVs can successfully infect the airway, model infection, and can be used to test interventions such as antiviral therapeies, passive or active immunization, and RBD blocking approaches. Finally, we believe that our system can seamlessly be extended beyond SARS-CoV-2 to model SARS-CoV, MERS-CoV, and future emerging coronaviruses.

There are potential angles where our model system could be improved for future use. One area is in the method used to introduce hACE2. While we believe that virus transduction of hACE2 is an attractive approach compared to hACE2 in transgenic mice because it allows for seamless adaptability in a variety of mouse strains, new transduction methods have emerged. In our study, AdV was used, but recent reports have demonstrated that AAV allows for sustained hACE2 expression for a longer period of time, theoretically expanding the subsequent PsV infection window, period of active infection, and intervention timeline [16]. However, truly elucidating how hACE2-AdV, hACE2-AAV, or hACE2tg systems alters SARS-CoV-2 pseudovirus infectivity requires empirical determination, and can be performed in our future studies. We utilized the D614G mutation, an amino acid change that mediates heightened infectivity and quickly became the most prevalent form of SARS-CoV-2 spreading during the COVID-19 pandemic, to enhance the infectivity of our PsVs. When exploring the ability of RBD protein administration to block infectivity of these PsVs, significantly less infection was observed in mice treated with RBD protein as compared to vehicle controls. It is important to note that WT RBD protein and 19del + D614G PsVs were used in this experiment, suggesting that WT RBD protein administration could have efficacy in preventing infection by some mutant SARS-CoV-2 PsV. The finding of our infection inhibition studies is consistent with that of other groups exploring the utility of soluble RBD-based proteins or anti-RBD antibodies to block SARS-CoV-2 infection [17, 18]. In our future studies, the utility of next-generation soluble RBD-based proteins, monoclonal anti-RBD antibodies, or serum from COVID-19 infected or vaccinated humans can be explored therapeutically in our system. Finally, although our PsV infection system is rapid and safe to employ, it does not fully recapitulate all aspects of SARS-CoV-2 infection. While we can study nasopharyngeal and lung infection using our PsVs, it is not adequate for investigating extrapulmonary infection and pathogenesis. In addition, our model provides a tool for studying viral entry, but may not be approporiate for studies on the post-entry events in the SARS-CoV-2 life cycle. Future studies on the development and engineering of SARS-CoV-2 PsVs and models should consider and aim to combat these limitations. 

## Conclusions

Our system of introducing hACE2 to the mouse respiratory system via AdV, followed by SARS-CoV-2 pseudovirus challenge is a simple, safe, effective method to model COVID-19 infection. SARS-CoV-2 PsVs can be adapted for in vitro use by employing using hACE2 cell lines. Therefore, this model can be used to test preventative and therapeutic strategies for the ongoing COVID-19 pandemic without the need for high biosafety levels or transgenic animal models. This model can potentially be used to model other coronavirus infections, giving it potential relevance for research on novel coronaviruses in the future.

## Supplementary Information

**Additional file 1: Fig. S1.** Detection of SARS-CoV-2 PsV infection in the lungs. Following establishment of infection as shown in Fig. 1c, mice were sacrificed on day 13 post infection following 5 min of i.n. D-luciferin exposure. Lungs were collected and imaged by IVIS. Representative images of 19del, 19del + D614G, or naïve lungs **(A)**

**Additional file 2: Fig. S2.** Titration of RBD-mediated blocking of PsV infection in vitro. As shown in Fig. 1f-h, additional 293TT cells were pre-treated with RBD one hour before addition of WT, 19del, or 19del + D614G SARS-CoV-2 PsVs. RLU detected in cell lysates 72 h after infection and blocking at the indicated RBD concentrations **(A)**.

## Data Availability

Data and materials are available from the corresponding author upon request.

## Abbreviations

COVID-19: Coronavirus Disease 2019
ACE2: Angiotensin-converting enzyme 2
hACE2: Human ACE2
AdV: Adenovirus vector
RBD: Receptor Binding Domain
VLP: Virus-like particle
ABSL-3: Animal biosafety level 3
PsV: Pseudovirus
i.n.: Intranasal
