# Supplementary figures and images for: A novel pseudovirus‐based mouse model of SARS-CoV-2 infection to test COVID-19 interventions
Source: J Biomed Sci. 2021 Apr 30;28:34. doi: 10.1186/s12929-021-00729-3 (PMC8084690; doi:10.1186/s12929-021-00729-3)

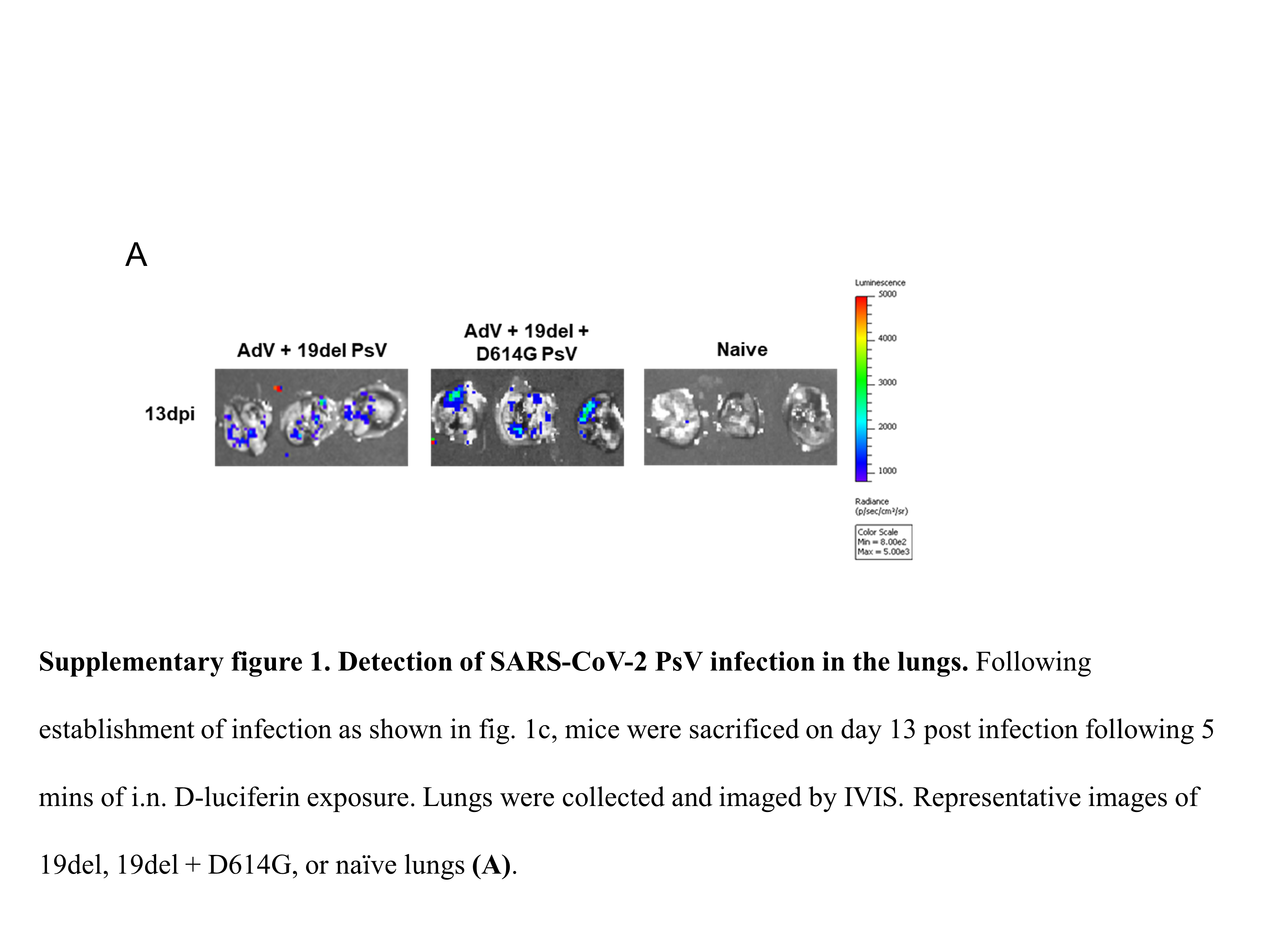

Supplement: Supplementary file 1 — Additional file 1: Fig. S1. Detection of SARS-CoV-2 PsV infection in the lungs. Following establishment of infection as shown in Fig. 1c, mice were sacrificed on day 13 post infection following 5 min of i.n. D-luciferin exposure. Lungs were collected and imaged by IVIS. Representative images of 19del, 19del + D614G, or naïve lungs (A) [file 12929_2021_729_MOESM1_ESM.tif]

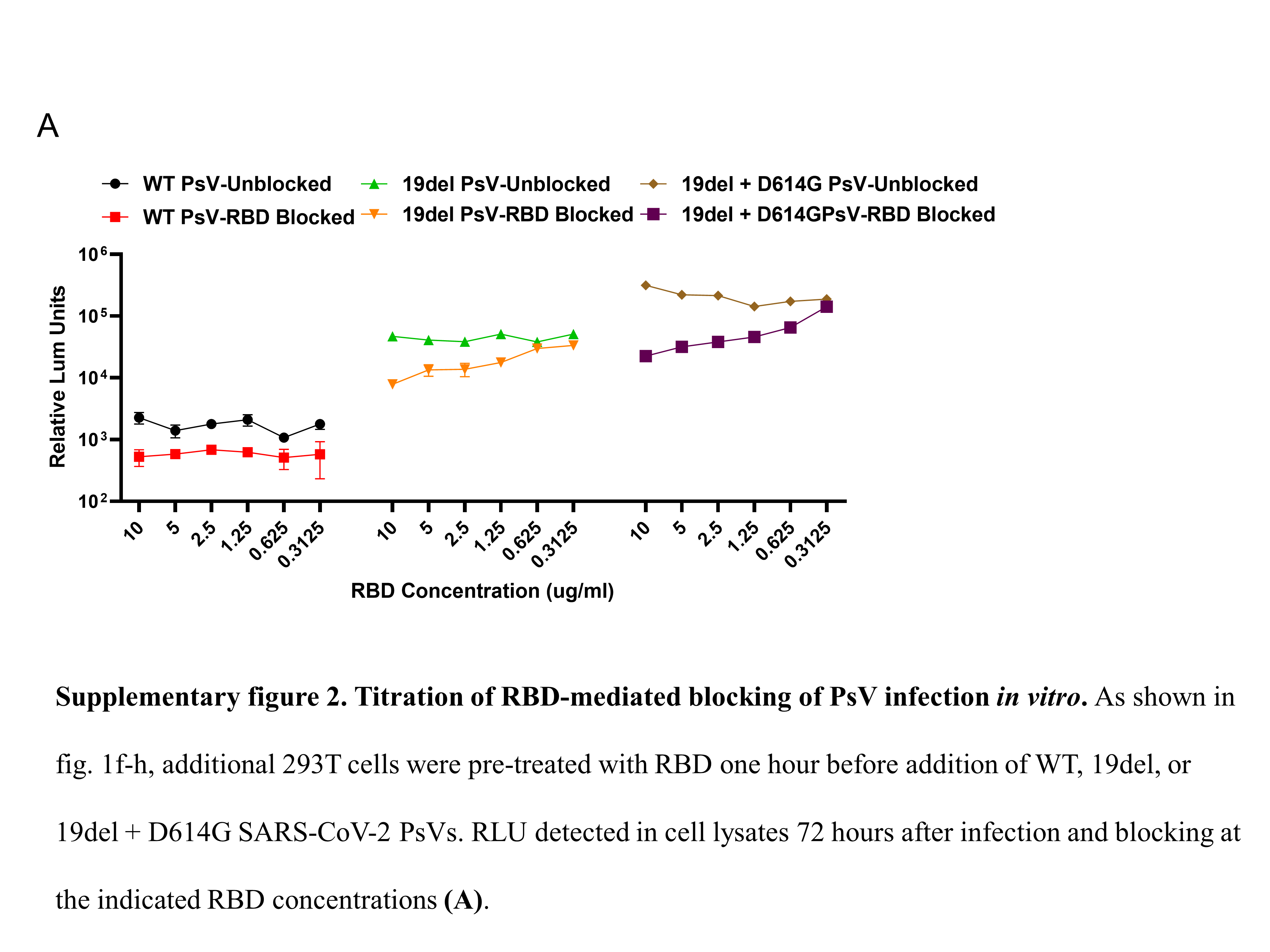

Supplement: Supplementary file 2 — Additional file 2: Fig. S2. Titration of RBD-mediated blocking of PsV infection in vitro. As shown in Fig. 1f-h, additional 293TT cells were pre-treated with RBD one hour before addition of WT, 19del, or 19del + D614G SARS-CoV-2 PsVs. RLU detected in cell lysates 72 h after infection and blocking at the indicated RBD concentrations (A). [file 12929_2021_729_MOESM2_ESM.tif]
